# The Probiotics in Pregnancy Study (PiP Study): rationale and design of a double-blind randomised controlled trial to improve maternal health during pregnancy and prevent infant eczema and allergy

**DOI:** 10.1186/s12884-016-0923-y

**Published:** 2016-06-03

**Authors:** Christine Barthow, Kristin Wickens, Thorsten Stanley, Edwin A. Mitchell, Robyn Maude, Peter Abels, Gordon Purdie, Rinki Murphy, Peter Stone, Janice Kang, Fiona Hood, Judy Rowden, Phillipa Barnes, Penny Fitzharris, Jeffrey Craig, Rebecca F. Slykerman, Julian Crane

**Affiliations:** Department of Medicine, University of Otago Wellington, PO Box 7343, Wellington South, 6242 New Zealand; Department of Paediatrics, University of Otago Wellington, PO Box 7343, Wellington South, 6242 New Zealand; Department of Paediatrics: Child and Youth Health, University of Auckland, Private Bag 92019, Auckland, 1142 New Zealand; Graduate School of Nursing, Midwifery and Health, Victoria University of Wellington, P O Box 7625, Newtown, Wellington 6242 New Zealand; Department of Obstetrics and Gynaecology, University of Otago Wellington, PO Box 7343, Wellington South, 6242 New Zealand; Dean’s Department, University of Otago Wellington, PO Box 7343, Wellington South, 6242 New Zealand; Department of Medicine, University of Auckland, Private Bag 92019, Auckland, 1142 New Zealand; Department of Obstetrics and Gynaecology, University of Auckland, Private Bag 92019, Auckland, 1142 New Zealand; Immunology Department, Auckland Hospital, Private Bag 92024, Auckland Mail Centre, Auckland, 1142 New Zealand; Murdoch Children’s Research Institute, The Royal Children’s Hospital, Flemington Road, Parkville, Melbourne, Victoria 3052 Australia

**Keywords:** Atopy, Infant eczema, Gestational diabetes, Postpartum depression, Pregnancy, Probiotic, Vaginal infections

## Abstract

**Background:**

Worldwide there is increasing interest in the manipulation of human gut microbiota by the use of probiotic supplements to modify or prevent a range of communicable and non-communicable diseases. Probiotic interventions administered during pregnancy and breastfeeding offer a unique opportunity to influence a range of important maternal and infant outcomes.

The aim of the Probiotics in Pregnancy Study (PiP Study) is to assess if supplementation by the probiotic *Lactobacillus rhamnosus* HN001 administered to women from early pregnancy and while breastfeeding can reduce the rates of infant eczema and atopic sensitisation at 1 year, and maternal gestational diabetes mellitus, bacterial vaginosis and Group B Streptococcal vaginal colonisation before birth, and depression and anxiety postpartum.

**Methods/design:**

The PiP Study is a two-centre, randomised, double-blind placebo-controlled trial in Wellington and Auckland, New Zealand. Four hundred pregnant women expecting infants at high risk of allergic disease will be enrolled in the study at 14–16 weeks gestation and randomised to receive either *Lactobacillus rhamnosus* HN001 (6 × 10^9^ colony-forming units per day (cfu/day)) or placebo until delivery and then continuing until 6 months post-partum, if breastfeeding.

Primary infant outcomes are the development and severity of eczema and atopic sensitisation in the first year of life. Secondary outcomes are diagnosis of maternal gestational diabetes mellitus, presence of bacterial vaginosis and vaginal carriage of Group B Streptococcus (at 35–37 weeks gestation). Other outcome measures include maternal weight gain, maternal postpartum depression and anxiety, infant birth weight, preterm birth, and rate of caesarean sections. A range of samples including maternal and infant faecal samples, maternal blood samples, cord blood and infant cord tissue samples, breast milk, infant skin swabs and infant buccal swabs will be collected for the investigation of the mechanisms of probiotic action.

**Discussion:**

The study will investigate if mother-only supplementation with *Lactobacillus rhamnosus* HN001 in pregnancy and while breastfeeding can reduce rates of eczema and atopic sensitisation in infants by 1 year, and reduce maternal rates of gestational diabetes mellitus, bacterial vaginosis, vaginal carriage of Group B Streptococcus before birth and maternal depression and anxiety postpartum.

**Trial registration:**

Australian New Zealand Clinical Trials Registration: ACTRN12612000196842.

Date Registered: 15/02/12.

**Electronic supplementary material:**

The online version of this article (doi:10.1186/s12884-016-0923-y) contains supplementary material, which is available to authorized users.

## Background

There is growing interest in the role the human microbiome has on the development of a range of chronic non-communicable autoimmune and inflammatory conditions [1]. It is now recognised that human gut microbiota can modulate the immune system with consequences expressed both inside and outside of the gut, including prevention or modification of risk for a range of allergic, autoimmune, cardiovascular and metabolic diseases [1, 2]. In addition, commensal gut microbiota have been demonstrated to modify host susceptibility to a range of infections [3]. Supplementation by probiotics is one approach used to intentionally modify the gut microbiota and thus influence health risks.

### Allergic diseases and probiotics

Worldwide, allergic diseases are the largest group of non-communicable diseases (NCD) with an increasing prevalence in both the developed and developing world [4]. They are also the NCD with earliest onset, children bearing much of the burden of these diseases [2]. The prevalence of atopic eczema has increased two to three fold in the last three decades with 15–30 % of children worldwide [4], and up to 40 % of infants in New Zealand having eczema by 15 months of age [5]. Sixty percent of children developing eczema will do so within the first year of life [4]. About half of the children who develop eczema early in life become sensitised to allergens by 2 years of age [4].

A significant body of research examining the use of probiotics to prevent allergic disease already exists. A meta-analyses of randomised controlled trials (RCT) shows benefits of using probiotic supplements during pregnancy and early infant life to prevent the development of atopic dermatitis [6]; however, a Cochrane review found that the benefit is not significant for immunoglobulin E (IgE) associated atopic dermatitis [7].

A more recent subgroup meta-analysis concluded that pre and post-natal supplementation is effective (OR = 0.61, 95 % confidence interval (CI) 0.52–0.71, *p* < 0.001) while there was no evidence for post-natal interventions-only being effective (OR = 0.95, 95 % CI 0.63–1.45, *p* = 0.82) [8]. Meta-analyses also indicate both treatments with *Lactobacillus* alone or *Lactobacillus* with *Bifidobacterium* appear to be protective (OR = 0.70, 95 % CI 0.54–0.89, *p* = 0.004; OR = 0.62, 95 % CI 0.52–0.074, *p* < 0.001) [8]. This is consistent with our own previous work in a RCT of 474 infants which showed that *Lactobacillus rhamnosus* HN001 (HN001) 6×10^9^ cfu/day given daily to mothers from 35 weeks gestation, continuing until 6 months post-partum if breastfeeding and from birth until 2 years in the infant was associated with a significant 50 % reduction in the prevalence of eczema at age 2 [9], 4 [10] and 6 years [11]. While not evident early, by 6 years there was also a significant reduction in skin prick sensitisation in the HN001 group (HR = 0.69, 95 % CI 0.48–0.99) [11].

Almost without exception [12] previous probiotic trials of allergic disease with a pre-birth intervention have commenced at some time in the final 2 months of pregnancy [6]. In our current trial we commence the intervention from 14 to 16 weeks gestation and continue it throughout pregnancy and for 6 months post-partum while breastfeeding, the probiotic being only given to the mother, not directly to the infant. Our justification for an early probiotic intervention is based on evidence showing that fetal production of IgE antibodies occurs before the end of the first trimester and allergen-specific IgE antibodies towards the end of the second trimester [13]. There is also evidence that maternal allergy alters the regulation of antigen-specific responses during pregnancy, with non-allergic mothers showing down-regulation of their (already lower) Th2 responses to specific allergen from mid to late gestation [14]. This down-regulation was absent in allergic mothers. Epidemiological support for the importance of intervention in early pregnancy comes from a longitudinal study which shows that maternal exposure to pollen during the first trimester of pregnancy increased the risk of food sensitisation in the child [15]. The majority of probiotic trials, using a late pregnancy intervention (from 32 to 35 weeks gestation), may therefore have missed the critical window to influence fetal immune responses and thus the later development of allergic disease. This may explain the general lack of effect of probiotics on infant sensitisation. The only study, by Huurre et al. [12], that did show a protective effect of probiotics on sensitisation in the infant used an early pregnancy intervention and the effect was limited to those with sensitised mothers (OR = 0.34, 95 % CI 0.13–0.88). In that study [12], the group of infants with non-sensitised mothers had a significantly *increased* risk of sensitisation but this finding was not reported in the article.

A RCT with a late pregnancy intervention [16] showed a reduction in child sensitisation also among children with allergic mothers (defined according to the presence of disease not atopic sensitisation). There was no effect among children of allergic fathers, highlighting the relative importance of the mother in influencing fetal immune development. In contrast, two probiotic trials [17, 18] have shown increased rates of sensitisation in all children taking probiotics but neither of these studies used *Lactobacillus rhamnosus,* and one intervened in infants only [18]. Confirmation of the role of probiotics in the development of atopic sensitisation in a larger study with an early pregnancy intervention may allow the targeting of a probiotic intervention to those who are most likely to benefit, i.e. in those with maternal sensitisation, while avoiding the possible increased risk of sensitisation among those without maternal sensitisation. As sensitisation is associated with more severe and persistent eczema [19], a probiotic intervention from early pregnancy, if found to protect against sensitisation, may also reduce the prevalence of clinically important eczema.

Previous pre and/or post-natal intervention studies also vary according to who received the probiotic intervention after birth: mother or infant or both. There have been two studies with an intervention only in the mothers (both from 36 weeks gestation and during breastfeeding) which have both shown an effect on eczema at 2 years that is as strong as that seen when the probiotics were also administered directly to the infant [20, 21]. Alteration in breast milk cytokine levels associated with allergic outcomes in those receiving probiotics suggest that immune modulation may also occur through this pathway [12, 21–23], and this indicates that post-natal maternal supplementation while breastfeeding may also be important. In contrast to many of the previous probiotic studies our current study administers the probiotics directly to women only, and if proven effective provides an intervention that is easier to administer as it does not require administration of probiotics to new-born infants. This would make the intervention more easily adopted into practice.

### Gestational diabetes and probiotics

Accompanying the worldwide trends in obesity, the rate of gestational diabetes mellitus (GDM) is also increasing in both the developed and the developing world [24]. Using the International Association of Diabetes and Pregnancy Study Group (IADPSG) [25] diagnostic criteria (fasting plasma glucose ≥5.1 mmol/l, or 1-hour post-75 g load ≥10.0 mmol/l, or 2-hour post 75 g load ≥8.5 mmol/l), 18 % of pregnant women in the United States develop GDM during pregnancy [24]. GDM is associated with short and long-term adverse outcomes for both women and infants, including maternal gestational hypertension, polyhydramnios, preeclampsia, delivery of large-for-gestation infants, instrumental or caesarean delivery, and maternal death [24, 26]. Adverse infant outcomes include preterm birth, shoulder dystocia, macrosomia, congenital defects, and neonatal complications such as hypoglycaemia, jaundice and respiratory distress [24]. In addition, in the longer term, women with GDM are at increased risk of metabolic syndrome [27], type 2 diabetes, and cardiovascular disease. Offspring of women with GDM have an increased risk of diabetes, obesity and metabolic issues with evidence of altered insulin secretion and lipid profiles regardless of the infant’s weight [28].

Lifestyle interventions to prevent GDM relating to diet, weight loss and exercise are often unsuccessful [24, 29]; therefore primary prevention of GDM could provide substantial multigenerational health and economic benefits. In a Finnish study [30, 31], among those receiving intensive dietary counselling, probiotic use (*Lactobacillus rhamnosus* GG and *Bifidobacterium lactis* Bb12 10^10^ cfu/day each) from the first trimester of pregnancy until the end of exclusive breastfeeding was associated with beneficial outcomes for GDM. The diagnostic test used in the Finnish study was a 75 g glucose OGTT with one value exceeding any of the following cut points being considered positive: fasting glucose value ≥4.8 mmol/l, or 1 h blood glucose ≥10.0 mmol/l, or 2 h blood glucose ≥8.7 mmol/l. Using these criteria, the prevalence of GDM was dramatically decreased, 13 % in women given dietary advice plus probiotics compared with 36 % in a group given dietary advice only and 34 % in a control group with no intervention (*p* = 0.003). The authors suggest that this effect may be due to probiotics contributing to glucose regulation during pregnancy [30]. In this same study population, probiotics taken from the first trimester were associated with half the risk of maternal adiposity, defined as having a waist circumference ≥80 cm, at 6 months post-partum (*p* = 0.03) [32]. A different study using *Lactobacillus rhamnosus GG* supplementation from 36 weeks gestation to 6 months postnatally to breastfeeding mothers or their infant found no significant change in birth weight adjusted mean BMI in the offspring at 4 and 10 years of age [33]. A more recent study [34] using a short probiotic intervention from 24 to 28 weeks gestation and different probiotic species (*Lactobacillus salivarius* UCC188 10^9^ cfu/day) in obese pregnant woman did not alter fasting glucose or other maternal outcomes. These findings may indicate that the probiotic species and strain as well as gestation at commencement of intervention, duration of intervention and concurrent diet contribute to the prevention of GDM. Our current study will examine the impact of HN001 supplementation from early pregnancy without altering baseline diet.

### Vaginal dysbiosis and probiotics

The maintenance of healthy vaginal microbiota is important for optimal pregnancy outcomes. Vaginal coliform and streptococcal colonisation occurs by intestinal microbes ascending from the perineum, and a healthy vaginal flora contains a predominance of organisms from the *Lactobacillu*s genus [35]. Lactobacilli protect the vagina from pathogenic organisms by producing antimicrobial agents such as hydrogen peroxide and bacteriocins, competing for nutrients, adhering to the epithelial surfaces, maintaining the vaginal pH through lactic acid production, and by immune modulation [36, 37]. Both bacterial vaginosis (BV) and Group B Streptococcus (GBS) colonisation are associated with depleted vaginal lactobacillus populations [38, 39] and are associated with negative pregnancy outcomes.

Internationally the prevalence of BV is high e.g. 25 % in pregnant women in USA [36]. BV is associated with preterm labour, premature rupture of membranes, spontaneous abortion, and chorioamnionitis [39]. Premature birth predisposes the infant to a range of other serious health issues including respiratory distress syndrome, intraventricular haemorrhage, leukomalacia, retinopathy, necrotising enterocolitis and prolonged hospitalisation with the associated costs to the health system [40]. Eighty percent of preterm deliveries result from premature rupture of the membranes and spontaneous preterm labour [40]. Maternal infections are associated with 30–50 % of preterm labours [40].

Antibiotic therapy (metronidazole) is recommended as treatment for BV, yet a large placebo-controlled trial did not find that metronidazole reduced the occurrence of preterm delivery or other adverse perinatal outcomes [41]. However a BV treatment study showed that a combination of orally administered *Lactobacillus rhamnosus GR-1 and Lactobacillus reuteri RC-14* and metronidazole doubled the cure rate compared to metronidazole alone [42]. Efficacy of current antibiotic treatments for BV is variable and recurrence is common (40 % at 3 months) [37]. In addition, antibiotic resistance in vaginal pathogens is an increasing concern [37]. Exploration of the role of probiotics in the prevention of BV-related adverse outcomes in pregnancy is in its infancy. A Cochrane review of probiotics for preventing preterm labour found an 81 % reduction in the risk of genital infection with the use of probiotics (RR 0.19; 95 % CI 0.08 to 0.48); however, there were insufficient trials to determine the effect on preterm birth and other complications [40]. Another review [43] of probiotics in the treatment and prevention of BV suggests a role for a range of *Lactobacillus* species in managing urogenital infections but studies with outcomes among pregnant women were absent.

GBS is a commensal bacterium found in the gastrointestinal and genitourinary tracts of 30 % of healthy adults [44]. Worldwide GBS vaginal colonisation in pregnant women varies with rates of between 4 and 36 % in European countries, and most countries having rates higher than 20 % [44–46]. Usually maternal GBS is asymptomatic, however it can cause endometritis, chorioamnionitis, and bacteraemia in pregnant women, and may cause stillbirth [47], and is the leading cause of early onset Group B Streptococcal septicaemia and meningitis in infants [44, 48]. Up to 50 % of babies born to colonised women acquire the infection and 1–2 % of colonised infants become seriously ill [49]. Despite the low rates of early onset infant GBS illness (1–4 cases/1000 live births) the consequences are potentially fatal, including sepsis, bacteraemia, pneumonia and meningitis with associated long term neurodevelopmental defects [44, 49]. Most countries use screening to detect vaginal GBS colonisation in pregnant women at 35–37 weeks. Those colonised receive intra-partum antibiotics to reduce the risk of vertical transmission to the infant during birth.

Lactobacilli have been shown to have inhibitory effects on GBS growth in vitro [50, 51] and vaginal *Lactobacillus* counts from pregnant women are inversely related to GBS colonisation [38]. Although the popular literature supports the use of probiotics in the prevention of GBS, there has been only one feasibility study examining an oral probiotic supplementation effect on GBS, and while non-blinded and not fully powered this study did find reduced GBS colony counts in the participants taking oral probiotic supplements [35].

Studies [43], including our own [9], have shown lactobacilli survive passage through the gastrointestinal tract, indicating that oral delivery of lactobacilli is feasible and may be expected to impact the composition of vaginal flora. In addition, HN001 has been demonstrated to produce bacteriocins [52], and appears not to have genes commonly associated with resistance to peroxide [GenBank Acc No. NZ_ABWJ00000000] so we anticipate that oral administration of this organism may favourably influence vaginal flora.

### Maternal postpartum depression and anxiety and probiotics

There is a growing literature on how gut microbiota might influence anxiety, depression and cognition via the microbiota-gut-brain axis [53–56]. Much of this work has been done in preclinical animal trials by intentional manipulation of the animal’s gut microbiota (such as using germ free mice, or treating with probiotics, antibiotics or pathogenic bacteria). Reviews of these studies demonstrate that alterations in anxiety-like or depressive behaviours in animals have been documented in response to manipulation of their gut microbiota [54, 56]. In particular, one study has shown that probiotic supplementation with *Lactobacillus rhamnosus* decreased anxiety-like and depressive-like behaviours in healthy mice [57].

A range of potential mechanisms by which the gut microbiota affects central nervous system function have been proposed. These include altered microbial composition, immune inactivation, vagal nerve activation, tryptophan metabolism, gut hormone response, and through production of neuro active substances or other metabolites [53, 56].

There is very limited published work describing mood or cognitive outcomes of probiotic interventions in humans. In a double blind, placebo randomised controlled trial; healthy subjects were given probiotics (*Lactobacillus helveticus* R0052 and *Bifidobacterium longum* R0175) or placebo for 30 days. The probiotic group had significantly less anxiety and depression than the controls [58]. In a similar study those subjects who initially scored in the lowest third for depressed mood showed significant improvement in symptoms after probiotic treatment [59].

A recent study provides the first direct evidence that probiotics alters brain activity in humans [60]. Daily intake of a mixture of four probiotic strains (*Bifidobacterium animalas* subsp *Lactis, Streptococcus thermophiles*, *Lactobacillus bulgaricus* and *Lactococcus lactis* subsp *Lactis*) over four weeks reduced brain activity to an emotional attention task in the regions of the brain that influence the processing of sensory information and emotion. Brain activity was assessed using functional magnetic resonance imaging. No changes in commensal bacteria were observed. The authors argued that the probiotics might interact with the host microbiota to alter their metabolic activity resulting in the production of metabolites that influence brain activity.

The prevalence of postpartum depression is variously estimated to be 10–15 % [61], and women with postpartum depression experience a range of symptoms including general anxiety and dissatisfaction with life, emotional lability, insomnia, confusion, guilt, and suicidality [62]. In addition maternal depression can interfere with mother-infant interactions and adversely affect the infant’s psychological and developmental trajectory at a time of vulnerability [62–64]. There have been no human studies examining whether probiotics given during pregnancy and breast feeding influence mood. With data emerging to indicate that probiotic effects may be mediated by the gut-brain axis, this study provides an excellent opportunity to assess this further by assessing postpartum depression and anxiety in our study population.

In summary the Probiotics in Pregnancy study aims to investigate if maternal supplementation by HN001 prevents infant eczema and atopic sensitisation by one year and improves maternal health during pregnancy by reducing GDM, BV and GBS, and in the early post-natal period by improving mood. The study design has an early maternal intervention at 14–16 weeks gestation continuing post-natally until 6 months after birth, if breastfeeding, which provides the unique opportunity to study these outcomes, all of which merit consideration on their own.

## Methods/design

### Study design and outcomes

The PiP Study is a two-centre randomised double blind placebo controlled trial in Wellington and Auckland, New Zealand designed to test the hypothesis that probiotic supplementation by *Lactobacillus rhamnosus* HN001 (6×10^9^ cfu/day) administered to mothers from 14 to 16 weeks gestation until delivery and continuing until 6 months post-partum, if breastfeeding, will reduce (1) the prevalence of infant eczema and atopic sensitisation by age 12 months and (2) the prevalence of maternal GDM, BV and GBS vaginal colonisation before birth. Table 1 provides an overview of primary, secondary and related outcomes being examined in the study.

**Table 1:** Primary, secondary and related outcomes in the PiP Study

Primary outcomes	Related outcomes
1. Infant eczema	Immune and inflammatory markers, epigenetic and metabolomic analyses from cord blood
	Epigenetic analyses on cord tissue
	Genotype and epigenetic analyses on buccal samples
	Immune markers and oligosaccharide profiles from breast milk
	Skin microbiota analysis from neck and axilla skin swabs and gut microbiota analysis from infant faeces
2. Infant eczema severity (SCORAD^a^)	
3. Infant atopic sensitisation	Immune and inflammatory markers, epigenetic and metabolomic analyses from cord blood
	Epigenetic analyses on cord tissue
	Genotype and epigenetic analyses on buccal samples
	Immune markers and oligosaccharide profiles from breast milk
	Skin microbiota analysis from neck and axilla skin swabs and gut microbiota analysis from infant faeces
Secondary Outcomes	Related Outcomes
1. Maternal gestational diabetes mellitus	Maternal:
	Gestation at delivery.
	Maternal anthropometric measures ^b^ at birth, and 6 and 12 months post-partum
	Caesarean section
	Fasting lipids and bile acids from fasting maternal blood taken at time of OGTT
	Gut microbiota and faecal SCFA from maternal faecal samples taken in second trimester
	Infant:
	Anthropometric measures at birth, and 6 and 12 months
	Neonatal intensive care (NICU) admission
2. Maternal BV	Premature rupture of membranes
	Preterm birth (<37 weeks)
3. Maternal vaginal GBS colonisation	Infant GBS
4. Maternal postpartum depression and anxiety	Medication use for emotional/psychological problems
Adverse events and safety	
1. Maternal:	2. Infant:
Anthropometric measures Bacteraemia/Septicaemia Hospitalisations (excluding birth admissions) Gastrointestinal symptoms Caesarean section rates	Anthropometric measures Bacteraemia/Septicaemia Hospitalisations (excluding birth admissions) 5 min Apgar scores Preterm birth Neonatal intensive care unit admissions Infant symptoms

^a^ *SCORAD* SCORing atopic dermatitis

^b^ Maternal height and head circumference recorded at 14–16 weeks gestation only

### Sample size and power calculations

#### Eczema

From our previous study the hazard ratio for eczema, for HN001, based on the data at 6 and 12 months, was 0.39, with 12.3 % of infants having eczema at either 6 or 12 months. With a sample size of 200 in each group and 13 % drop-out rate, 43 babies are expected to be assessed as having eczema. Using the sample size formula from Therneau and Grambsch [65], the study will have 87 % power at the 5 % level of significance.

#### SCORing Atopic Dermatitis (SCORAD)

Our previous study [9] found a reduction in SCORAD [66] ≥10 from 22.9 to 12.8 % due to the probiotic intervention. With a sample size of 200 in each group and a 13 % drop-out rate, the study will have 69 % power at the 5 % level of significance.

#### Atopic sensitisation

In general, effects of probiotics on sensitisation have only been seen in a subset of children with allergic [16] or sensitised mothers [12]. Assuming 60 % of mothers are skin prick test positive and a reduction in the prevalence of atopy from 50 to 26 % among their children, as seen in the paper by Huurre et al. [12], with a sample size of 200 pregnant women (120 with mothers who are sensitised) in each group and 13 % drop-out rate our study will have 95 % power at the 5 % level of significance, for atopic sensitisation among the children of mothers that are skin prick test positive. A further analysis of the data from the study by Huurre et al. [12] shows that there is a four-fold increased risk of sensitisation in the sub-group without maternal sensitisation. Assuming a similar difference in risk dependent on mother’s atopic status, the study would have >99 % power to show an interaction effect.

#### Gestational diabetes mellitus

Assuming a GDM prevalence rate of 15 %, and based on the study by Luoto [31] which showed a 63 % reduction in prevalence of diabetes mellitus in pregnant women due to a probiotic intervention, with a sample size of 200 in each group and a 2.5 % drop-out rate the study will have 87 % power to detect a difference at the 5 % level of significance.

#### Bacterial vaginosis

We cannot locate any published data on the prevalence of BV in New Zealand women but international data suggest it is around 30 % [67]. A probiotic treatment RCT of BV by Anukam [42] found a 88 % cure rate (defined as normal nugent score and other markers of BV at 30 days post treatment) in the probiotic group and 40 % in the placebo group. Assuming a 30 % rate in the placebo group, and a similar probiotic effect resulting in a 6 % rate of BV in the probiotic group, the study will have >95 % power at the 5 % level of significance with a sample size of 200 in each group and 2.5 % drop-out rate.

#### Group B streptococcus

We are not aware of any studies of a probiotic treatment effect on GBS carriage on which to base power calculations but the New Zealand prevalence was reported to be 22 % in 1998-9 [46]. With a sample size of 200 in each group and a 2.5 % drop-out rate, the study will have 84 % power to detect a reduction to 11 % at a 5 % level of significance.

### Maternal postpartum depression and anxiety

The distribution of the Edinburgh Postnatal Depression Scale (EPDS) from Growing up in New Zealand Study [68] was used. With a sample size of 200 in each group and 13 % drop-out rate, using a Wilcoxon Rank-Sum test, the study will have 79 % power to detect a 26 % reduction in EPDS at a 5 % level of significance.

## Ethics

The study received ethical approval from the New Zealand Multi-region Ethics Committee (MEC/11/09/077). Prior to commencing the study, adult participants receive all the necessary information in relation to the study, and written informed consent is obtained. Women provide written informed consent on behalf of both themselves and their unborn child/infant.

### Study population

#### Pregnant women

English-speaking pregnant women who intend to breastfeed their infant are eligible to be enrolled in the study between 14 and 16 weeks gestation if either they or the unborn child’s biological father have a history of asthma or eczema treated by a doctor, or allergic rhinitis treated by a doctor or pharmacist. A woman is excluded if she: is under age 16, does not intend to stay in either of the study centers for the 18 months following enrolment, has a serious immunological disorder that suppresses immune function or is taking immune suppressant drugs, has known cardiac valve disease for which antibiotic prophylaxis is required when undergoing dental procedures, has a history of a transplant or human immunodeficiency virus, were on long-term of continuous antibiotic therapy, required in vitro fertilisation to establish the current pregnancy, has a pre-enrolment scan showing major fetal abnormalities, at the time of enrolment is using or intended to use probiotic drinks or supplements themselves or in their child, is participating in another randomised controlled trial, has a severe allergy to cow’s milk (as probiotic capsules may contain traces of milk), has previously participated in the study with an older child or is deemed unsuitable for study inclusion for any other medical reason. Any pregnant woman with pre-existing type 1 or 2 diabetes is eligible for the study but is excluded from the oral gestational tolerance test (OGTT) and gestational diabetes outcomes.

#### Infants

All infants born in the study are eligible for inclusion in study outcomes. In the case of multiple births, only the first-born infant will be included in the study.

#### Fathers

The biological father of the infant is invited to participate in a study visit to obtain some background data relating to their history of allergic disease, atopic sensitisation by skin testing and anthropometric measures. These data are being collected to enable examination of the relationship between paternal characteristics and infant growth, allergic disease and atopic sensitisation.

### Recruitment

A range of recruitment strategies are used to notify pregnant women about the study and to invite them to contact the study centres to discuss the study and be assessed for study eligibility. These include: enclosing study brochures in information packs routinely given to women by clinicians and midwives in early pregnancy, emailing employees of large organisations, using web-based approaches such as Facebook, arranging for sonographers and phlebotomists to hand out study brochures at the time of early scans and the first antenatal blood collection, radio advertising, newspaper articles as well as pamphlets and posters placed at hospitals, clinics, crèches, libraries and pharmacies and inviting clinicians (midwives, obstetricians and general practitioners) to refer women to the study.

Pregnant women who contact the study centres are screened for eligibility by phone and if eligible are invited to attend an enrolment visit at the study centre when they reach between 14 and 16 weeks gestation.

### Study intervention

#### Capsules

Study capsules containing *Lactobacillus rhamnosus* HN001 (6x10^9^ cfu) are manufactured by Fonterra Co-operative Group Ltd as previously described [9]. Shelf life is managed to ensure minimum viable counts of 6×10^9^cfu are maintained in the HN001 capsules. The placebo powder is corn-derived maltodextrin, manufactured by Grain Processing Corp. Oregon, USA and is supplied to Fonterra Co-operative Group Ltd by Salkat New Zealand Ltd, Auckland. Placebo capsules have identical appearance and smell to the probiotic capsules. Both probiotic and placebo powders are encapsulated by Alaron Products Ltd, Nelson, New Zealand and provided in opaque bottles. Quality and safety testing is performed to a pharmaceutical standard (Therapeutic Goods Act) by a registered external laboratory.

#### Randomisation process

Randomisation is managed by Fonterra Co-operative Group Ltd and concealed from all study staff and participants. Randomisation is stratified by study centre and performed in blocks of random lengths according to a computer-generated randomisation list. At enrolment, a research staff member assigns the woman the next consecutive study number and provides her with the appropriate capsules.

#### Capsule viability and compliance

All capsules are stored at 4° Celsius (°C) in the study centres and participants are advised to store the capsules in their home refrigerator. Written and verbal instructions regarding use and storage of capsules are provided to participants. Women are advised to take one study capsule daily from enrolment until 6 months after birth if breastfeeding, or to cease capsules before 6 month post birth if they have not breastfed for 24 h. To assess compliance, capsule bottles are collected at regular intervals and counts of remaining capsules are completed by an independent person. Samples of capsules returned from the field are tested to ensure that capsules have maintained their viability. As previously described [9], viable bacteria are counted using standard methods, and counts checked against samples of HN001 kept at 4 °C.

### Study assessments

Table 2 provides a summary of the study timeline and investigations. Women enrolled in the study are provided with a study calendar to assist them to prospectively record data relevant to the study questionnaires. Research staff members are trained in standardised administration of all study questionnaires and data collection procedures.

**Table 2:** Study timeline and investigations

		14-16 Weeks Gestation	26-28 Weeks Gestation	35-37 Weeks Gestation	Birth	3-7 Days After Birth	Infant Age 3 Months	Infant Age 6 Months	Infant Age 12 Months
	Intervention: new bottle study capsules	✓ Intervention commences	✓			✓	✓	Intervention stops	
	Questionnaire	✓	✓			✓	✓	✓	✓
Objective measures and samples: Maternal									
	Vaginal swab (BV)	✓		✓					
	Vagino-rectal swab (GBS)	✓		✓					
	Anthropometry^a,b,c,d^	✓				✓		✓	✓
	Blood samples (OGTT and serum)		✓^e^						
	Faecal sample		✓						
	3 day food diary		✓						
	Cord blood and tissue				✓				
	Breast milk					✓			
	Skin prick test							✓Completed after birth	
Objective measures and samples: Infant									
	Anthropometry^a,b,c^					✓		✓	✓
	Eczema							✓	✓
	SCORAD							✓	✓
	Faecal sample								✓
	48 hour food diary								✓
	Buccal sample								✓
	Skin swabs								✓
	Skin prick test								✓
Objective measures and samples: Paternal									
	Anthropometry^a,b,c^					✓Completed at any visit after birth			
	Skin prick test					✓Completed at any visit after birth			

*BV* bacterial vaginosis, *GBS* group B streptococcus, *OGTT* oral glucose tolerance test, *SCORAD* scoring atopic dermatitis

^a^height/length ^b^weight ^c^head circumference ^d^waist circumference ^e^excluding women with pre-existing diabetes

Questionnaires collect a range of data including confounders (such as previous GDM, parity etc.), effect modifiers (such as use of antibiotics, yoghurt etc.), outcome data, and adverse events.

#### Assessment of primary outcomes

##### Infant eczema

After withholding topical creams and oils for 24 h the prevalence of eczema in infants is assessed at 6 and 12 months using the UK Working Party’s Diagnostic Criteria for atopic dermatitis [69] which is adapted for infants under 12 months [9]. All research staff members undertake online training in the application of this tool. Eczema is assessed as present at each visit if there is a history of scratching or rubbing and two or more of the following occurring since birth or the previous visit: (1) a history of involvement of outer arms or legs, (2) a history of a generally dry skin, or (3) visible eczema present on the cheeks or outer arms or legs with no axillary involvement. Eczema severity is assessed at the same time points using SCORAD [66] with staff recording any region of greater than or equal to 1 cm diameter that meets the skin change criteria [69] for eczema. For the purpose of consistency between the UK Working Party’s definition of atopic dermatitis and SCORAD, papulation is considered a surface change when using these tools.

##### Atopic sensitisation

Skin prick tests are performed using standardised protocols, (after relevant antihistamine withhold periods if needed) using Stallergenes 1 mm lancets (Antony, France), positive control (histamine 10 mg/ml), negative control (phenolated glycerol-saline), *Dermatophagoides pteronyssinus*, cat fur, five grass mix (cocksfoot, sweet vernal, rye, timothy and meadow grass), (Alyostal Stallergenes, Antony, France), egg white, peanut and cow’s milk (ALK- ABELLO, Madrid, Spain). Both biological parents are tested with positive, negative controls and aeroallergens only. Infants are tested at 12 months using both controls and all allergens. After application allergens are pricked vertically for 1 s, positive control wheals are measured at 10 min, and the negative control and all allergens are measured at 15 min to the nearest mm. The wheal size is calculated as the mean of the longest diameter and the midpoint perpendicular diameter. After the subtraction of any reaction to the negative control a mean wheal diameter of 3 mm or greater will be considered a positive reaction.

#### Assessment of secondary outcomes

##### Gestational diabetes mellitus

Women who are diagnosed with diabetes prior to 20 weeks gestation are excluded from the gestational diabetes outcomes for the study, as this diagnosis is likely to reflect pre-existing diabetes. Gestational diabetes outcomes are assessed at 26–28 weeks gestation, by a three time-point test 75 g oral glucose tolerance test (OGTT) recommended by international guidelines [25]. Preparation for the test include a 10–16 h pretest fast with instructions to avoid smoking during this time and during the test, and avoidance of exercise on the morning of the test. Participants remain in a resting state during the test which comprises of a baseline fasting blood sample, 75 g glucose load and blood samples at 1 and 2 h post load. An additional blood sample is collected at the time of the fasting blood test and this sample is separated and the serum stored at −80 °C for future analysis.

##### BV and GBS

All swabs and blood samples are processed using standard laboratory practices by accredited medical laboratories in the study centres. Vaginal swabs for BV and vagino-rectal swabs for GBS are self-collected by woman at enrolment (baseline) and 35–37 weeks gestation (outcome) following standardised written instructions and using Copan Transystem Amies (Ref 108CF.FR) swabs. Additional file 1: Figure S1 shows the swab collection instructions in more detail. After collection, swabs are transported at ambient temperature to laboratories as soon as is possible but within a maximum of 24 h. BV swabs are gram stained, and reported according to the three bands of Nugent’s scale, (0–3 being normal, 4–6 intermediate, and 7 or more indicating BV) [70]. GBS swabs are reported according to the presence or absence of GBS. As GBS is not treated in early pregnancy and BV is only treated if symptomatic, the results of baseline swabs remain confidential to the study. This is to ensure study swabs do not unintentionally result in unrequired antibiotic treatment being prescribed by reporting positive swabs which may not be from symptomatic disease. Only the results of the outcome 35–37 week GBS swabs are shared with the woman’s lead maternity carer (LMC)^1^ as these swabs inform the woman’s clinical care.

##### Maternal postpartum depression and anxiety

At 12 months post birth or later women will be asked to provide retrospective information related to their mood at 1–2 months after birth. Questions include the EPDS [71], state anxiety inventory [72], with the addition of questions to collect medication use for emotional and psychological problems.

#### Other measures

##### Anthropometric measures

Anthropometric measures are being performed using regularly calibrated standardised equipment and according to protocols adapted from the World Health Organisation [73] (see Additional file 2: Table S3).

##### Food diaries and faecal samples

Three day food diaries and faecal samples are collected from women at the time of their OGTT, and infants at 12 months. Participants are requested to prospectively record all foods and drinks taken during the diary period including the amounts taken, methods of cooking, any additions to the food (e.g. sauces) and any dietary supplements taken during the period. The food dairy is completed in the 2–3 days immediately prior to the collection of a faecal sample.

Faecal samples are collected into clean containers, then a small portion of faecal material is immediately transferred into hospital grade faecal sample pots and frozen at −20 °C with the sample container surrounded by water to ensure the sample remains frozen while being transferred to the research centre. Samples are collected no more than 48 h before being transferred to the research centres and stored at −80 °C. A range of quality control data are collected regarding these samples, detailed in Additional file 3: Table S4.

##### Cord blood and tissue samples

Cord blood and cord tissue samples are collected from participants in Wellington only. These samples are being collected for a range of studies to elucidate the mechanisms of probiotic action including cytokine, epigenetic and metabolomic studies (see Table 1). Using standardised instructions, intra-partum carers collect up to 40 ml of cord blood using an 18 gauge needle and sterile syringe, and 2 cord tissue samples. Further details of sample collection, processing and quality control data are presented in Additional file 4: Table S5.

##### Breast milk samples

Pre-feed breast milk samples are collected by women at 4–7 days after birth after the breast milk has come in. Women are advised to wash their hands prior to sample collection, then express a few drops of milk and discard before expressing a 10–15 ml into a sterile polypropylene 70 ml container. This sample is divided between three 5 ml sterile leak-proof freestanding polypropylene polyethylene Rnase and Dnase free tubes (Interlab code 3811-345-008). Samples are chilled in the home refrigerator if the sample will be picked up by research staff within 48 h or frozen in the home freezer with water surrounding the milk tubes if the sample will be picked up more than 48 h after collection [74]. All samples are stored at −80 °C. Quality data collected for these samples are documented in Additional file 5: Table S6.

##### Infant skin swabs

To enable examination of the infant skin microbiota two skin swabs are taken, one from the infants left neck (a common site for eczema) and another from the left axilla (a site where eczema is uncommon) at the 12 month visit using protocols adapted from the manual of procedures from the Human Microbiome Project [75]. Prior to sampling, parents are advised to avoid the following: antibiotic or steroid creams for 7 days (if possible), antiseptic or antimicrobial soaps or bath additives for 48 h, swimming for 48 h, washing for 24 h, and any creams or oils on the skin on the day of the visit. Questionnaire data is obtained to assess compliance with these preparations and to collect data related to any antibiotic use which may alter swab outcomes. To avoid possible contamination of samples with the researcher’s skin microbiota, these samples are collected at the commencement of the study visit and prior to the researcher having other physical contact with the infant. Samples are collected using sterile gloves and aseptic technique and Eswabs containing Amies fluid (480CE ESwab LQ Amies Reg. Nylon Flocked Applicator, Copan). Neck swabs are taken from the left side of the neck midway down a vertical line from earlobe to base of neck. The swab is moistened in Amies fluid and then using firm pressure and covering an area of 4 cm^2^ swabbed back and forth five times (one complete swab of back and forth is counted as one cycle). The swab is then rotated so the opposite swab surface is touching child’s skin and rubbed back and forth again in the same area for a further five cycles. A second swab is taken from the left axilla in the same manner. Following collection, swabs are immediately placed on ice prior to storage at −80 °C. Quality data includes date and time of swab collection and placement at −80 °C, and if full 10 cycles of swabbing is completed.

##### Infant buccal sample

Two infant buccal samples are taken at 12 months to enable future genotyping and epigenetic analysis. To prevent sample contamination parents are advised to avoid giving their child anything other than water to eat or drink, and to avoid cleaning teeth in the hour prior to sample collection. Research staff wear clean gloves and samples are collected using sterile Copan 502CS01 regular flocked swabs and by rubbing up and down 10 times against the inside of the cheek then gently rubbing in the grooves next to the top and bottom gums on the same side for 10 s. Swabs are placed in sterile 5 ml sterile SIMP309-5A cryovials and immediately placed on ice until transferred to a −80 °C freezer. Date and time of swab collection and time of placement at −80 °C are recorded. The child’s grandparents and mother’s ethnicity are recorded.

### Safety and adverse event monitoring

Our previous study using HN001 in women from 35 weeks gestation until 6 months post birth and in their infants for 2 years found no adverse effects [76]. Similarly other studies of safety using differing strains of *Lactobacillus rhamnosus* [77], and studies with interventions commencing from the first trimester [31, 78] have found no adverse effects on maternal (gestation length, miscarriage, caesarean delivery) or infant (Apgar score, birth weight and length, head circumference) outcomes.

Adverse event data are collected in all questionnaires. In addition, study doctors review the notes of any participant where there is any event that may require review, such as if the infant is delivered at less than 37 weeks’ gestation; if an infant requires resuscitation at birth; if the infant has any malformations (apart from tongue tie); if the infant requires admission to neonatal unit for more than 48 h for any reason; if the woman or baby had any hospital admission other than the birth admission; if the women or infant have any serious infection (e.g. septicaemia) or any other serious health issues.

### Data analysis

An intention-to-treat analysis will be used to assess the effect of HN001 on the 12 month cumulative prevalence of eczema and SCORAD ≥10 using hazard ratios from a Cox’s proportional hazards model, and the point prevalence of atopy at age 12 months using relative rates and a chi-squared test. Chi-square test will also be used to compare the proportion in each study group with detectable levels of immunological markers in breast milk and for those with detectable levels, t-tests will be used to compare the groups if the data is log-normally distributed. If the data is not log-normally distributed a Wilcoxon Rank-Sum test will be used. Where there are important differences between groups, these variables will be added to the models to assess whether these variables mediate any effect of HN001 on eczema and/or atopic sensitisation (using a generalised linear model with a log link and binomial distribution). The association of HN001 with the presence of GDM, BV and GBS will be assessed using relative rates and chi-squared tests. EPDS and State Anxiety Inventory will be compared with a Wilcoxon Rank-Sum test. The effect of weight gain will be investigated as an intermediate variable by adding it to the model (a generalised linear model with a log link and binomial distribution) in the GDM analysis. Any baseline differences that occur will be adjusted for. If commercially available non-study probiotics have been used, a sub-group analysis will be performed after excluding this group, which we do not expect to be large. Reasons for withdrawal from the study will be recorded and examined for differences between study groups. Adjustment will be made for differences in antibiotic use between study groups. SAS version 9.4 will be used.

## Discussion

In spite of the growing body of evidence for the benefits of probiotics in prevention of eczema these interventions have not been adopted into routine clinical practice [79]. The heterogeneity of primary prevention studies with different combinations of probiotics, strains, doses and timeframes for interventions makes meaningful synthesis of studies difficult. A recent 2015 World Allergy Organisation guideline has for the first time suggested that pregnant women whose infants are at high risk for allergy should take probiotics; however this recommendation is recorded as conditional and based on very low quality evidence [80]. In spite of the need for more research in this area, undertaking such studies is complex. Commercially available probiotic supplements are increasing in number and are readily accessible. In conjunction with this we believe that there is an increasing community acceptance and confidence in the benefits probiotic supplementation provide. Recruitment to the PiP Study is slower than originally planned partly because some eligible pregnant women decline study enrolment because they did not wish to be randomised to receive the placebo, choosing instead to purchase their own probiotic supplements.

We routinely discourage use of probiotic supplements other than the study capsules, but so we can account for any non-study probiotic exposure, we record details of any that are taken. We also collect data on use of yoghurts which are the most commonly consumed food sources of probiotics in New Zealand. We notice an increasing array of foods and drinks on the market which contain probiotics, and while many of these may be inactive or have minimal effectiveness such exposures are not easily incorporated into study data collection. These issues create significant challenges in the process of building a reliable and strong evidence base to enable confident recommendations to be made in clinical settings.

In comparison with our previous work with HN001 [9–11] showing a protective effect for the development of eczema the design of this study changes several things. This includes the different timeframe for the commencement of the intervention (14–16 weeks gestation compared to 36 weeks gestation previously), with only the women receiving the probiotic supplementation (compared to both women and infant previously), and final allergic outcomes will be measured at age 12 months (compared to 24 months previously). If this study does not demonstrate a protective effect for development of eczema it will be difficult to interpret which of these factors has caused a change in effect.

### Clinical significance

The PiP Study will assess the efficacy of HN001 supplementation from 14 to 16 weeks gestation in the prevention of GDM, BV, and vaginal GBS colonisation in pregnant women, and development of eczema and atopic sensitisation in their infants at one year and maternal depression and anxiety at 1–2 months postpartum. A unique strength of the study is the range of maternal and infant outcomes examined as a result of a single intervention. If successful the intervention could be adopted into practice with relative ease. This is in contrast to other probiotic eczema prevention strategies which also require direct infant supplementation. Through analysis of biological samples collected prospectively the study may also provide insights into the mechanisms of probiotic action in prevention of gestational diabetes, infant eczema and infant atopic disease. In addition the study will provide further valuable information of the safety of the HN001 supplementation in these populations.

### Trial status

The PiP Study is ongoing, and final outcome data are due to be collected by mid-2016.

## Abbreviations

BV, bacterial vaginosis; °C, degrees Celsius; cfu, colony-forming units; cfu/day, colony-forming units per day; EPDS, Edinburg postnatal depression scale; GDM, gestational diabetes mellitus; GBS, group B streptococcus; HN001, *Lactobacillus rhamnosus* HN001; NCD, non communicable diseases; OGTT, oral glucose tolerance test; PiP Study, Probiotics in Pregnancy Study; RCT, randomised controlled trial; SCFA, short chain fatty acids.

## Additional files

Additional file 1: Figure S1. Instructions for taking vaginal and vagino-rectal swabs for the Probiotics in Pregnancy Study. Description: Detailed instructions for study participants to take vaginal and vagino-rectal swabs. (PDF 165 kb) Additional file 2: Table S3. Anthropometric measures: equipment and protocols. Description: Equipment and protocols for anthropometric measures. (PDF 37 kb) Additional file 3: Table S4. Quality data for faecal samples. Description: list of quality control data recorded for infant and maternal faecal samples. (PDF 30 kb) Additional file 4: Table S5. Cord blood and tissue collection, processing and quality data. Description: Description of collection equipment, laboratory processing and quality control data for cord blood and tissue samples. (PDF 36 kb) Additional file 5: Table S6. Quality data for breast milk samples. Description: Details of data recorded for quality control of breast milk samples. (PDF 30 kb)

## Footnotes

1 In New Zealand, maternity care and delivery is managed through a system of lead maternity carers. These carers provide maternity care and support through the pregnancy, co-ordinate with other healthcare providers, organise scans and tests, manage the labour and provide care for the first six weeks of the baby’s life. Most lead maternity carers are midwives, though some doctors and obstetricians may also carry out the role.
