# Supplementary material for: The Probiotics in Pregnancy Study (PiP Study): rationale and design of a double-blind randomised controlled trial to improve maternal health during pregnancy and prevent infant eczema and allergy
Source: BMC Pregnancy Childbirth. 2016 Jun 3;16:133. doi: 10.1186/s12884-016-0923-y (PMC4891898; doi:10.1186/s12884-016-0923-y)
Supplement: Additional file 1: Figure S1. — Instructions for taking vaginal and vagino-rectal swabs for the Probiotics in Pregnancy Study. Description: Detailed instructions for study participants to take vaginal and vagino-rectal swabs. (PDF 165 kb) [file 12884_2016_923_MOESM1_ESM.pdf]

Figure 1: Instructions for taking vaginal and vagino-rectal swabs for the Probiotics in Pregnancy Study

1. Wash and dry your hands.
2. Place the swab packets side by side on a stable clean surface.
3. Place each colour coded laboratory label next to the matching coloured packet.

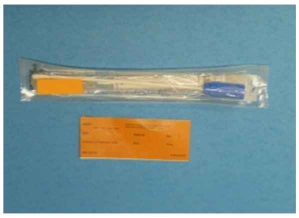

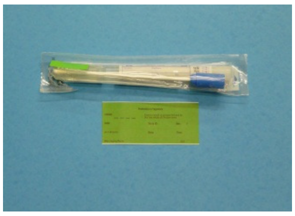

4. Peel apart JUST THE END of the plastic wrapping of both swab packets and keep the swabs and tubes in their plastic wrap.
5. Pull the white caps off the tubes and discard the caps into rubbish bin.

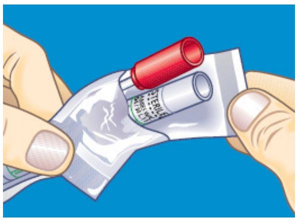

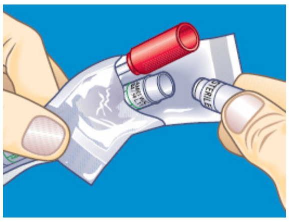

6. Take the swab stick from the ORANGE packet and hold it ONLY by the RED/BLUE cap.
7. NEVER touch the white stick as this will contaminate the swab.

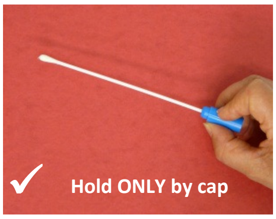

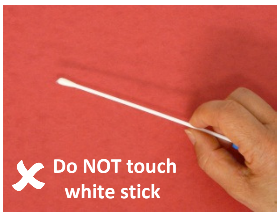

8. Sit or stand in a comfortable position. Some women prefer to stand with one leg raised on the toilet seat while others prefer to squat down.

Vaginal swab

9. Hold the swab from the ORANGE packet in one hand. With your other hand, gently part the skin folds at the vaginal opening.
10. Insert the swab into your vagina and direct it towards your lower back. This is similar to the way you would insert a tampon.
11. Stop when you have inserted the stick half its length.
12. Gently turn it around 3 times.
13. Remove the swab and put it in the tube. Push the RED/BLUE cap on tightly.
14. Keep the tube inside the ORANGE packet.

Vaginal/Rectal Swab

15. Take the swab from the GREEN packet and repeat the process as you did with the vaginal swab.
16. Immediately after removing the swab from your vagina, pass the tip of the same swab to your anus (back passage). Insert the tip gently 1 cm into the anus.
17. Remove the swab and put it in the tube. Push the RED/BLUE cap on tightly.
18. Keep the tube inside the GREEN packet.

17. Wash and dry your hands again.

18. Remove the tube from the ORANGE packet and stick the ORANGE label onto it.

19. Remove the tube from the GREEN packet and stick the GREEN label onto it.

20. Place the tubes in the zip-lock section of the laboratory bag.
21. Fill in the date and time you took the swabs on the laboratory form. Put the form into the side pocket of the bag.
22. Wash your hands again
23. Give the bag to the study nurse.
