# Supplementary material for: The Probiotics in Pregnancy Study (PiP Study): rationale and design of a double-blind randomised controlled trial to improve maternal health during pregnancy and prevent infant eczema and allergy
Source: BMC Pregnancy Childbirth. 2016 Jun 3;16:133. doi: 10.1186/s12884-016-0923-y (PMC4891898; doi:10.1186/s12884-016-0923-y)
Supplement: Additional file 2: Table S3. — Anthropometric measures: equipment and protocols. Description: Equipment and protocols for anthropometric measures. (PDF 37 kb) [file 12884_2016_923_MOESM2_ESM.pdf]

Table 3: Anthropometric measures: equipment and protocols

	Adult	Infant	Acceptable variance
**Head ciirc.**	Equipment: Seca tape measure 201 1717009.<br>1. Measurement: the maximum diameter from the bony ridge located above the eye sockets and placed posteriorly around the widest part of the head.	Equipment: Retractable tape measures from Pfizer Nutrition.	1. Measure to the nearest 0.1 cm.<br>2. Repeat measures until two measurements are within 0.3 cm.
**Height/Length**	Equipment: Marsden Height measurement Manufactured by Charder Electronic Company Ltd.<br>1. Measure without shoes, and take down any hairstyle which may interfere with the headboard making contact with the vertex of the head.<br>2. Subject stands on the base plate with weight distributed evenly on both feet, heels together and head positioned looking straight forwards. Arms hang freely at sides.<br>3. Subject's body should not be in contact with the stadiometer upright so as not to deflect it from vertical.<br>4. Subject stretches to fullest height.<br>5. Bring the headboard down to make contact with the vertex of the subject's head.	Equipment: Baby length measure map Seca 210 1821009.<br>1. The infant lies in a supine position on a recumbent measuring mat. The crown of the head must touch the stationary, vertical headboard.<br>2. Align infant head so the line of vision is perpendicular to the plane of the measuring surface.<br>3. The shoulders and buttocks must be flat against the measuring surface, with the shoulders and hips aligned at right angles to the long axis of the body.<br>4. The legs must be extended at the hips and knees and lie flat against the surface and the arms rest against the sides of the trunk.<br>5. Ensure that the legs remain flat on the surface and bring the movable board against the heels to measure.	1. Record the measurement to the nearest 0.1cm.<br>2. Repeat measures until two measurements are within 0.5cm.
**Weight**	Equipment: Tanita personal scales HD-316 (Manufactured by Wedderburn) or Salter Bathroom scales Model 9175.<br>1. Ensure subject is wearing only light clothing.<br>2. Place scales on a hard surface (not carpet).<br>3. Activate the scales and 'zero'.<br>4. Subject stands on the centre of the scales, with the body weight evenly distributed between both feet.<br>5. Subject remains still until the scales have stabilised on a reading.	Equipment: Soehnle 8310 infant scales.<br>1. Place scales on a firm surface (not carpet).<br>2. Zero the scales and then weigh infants naked OR<br>3. Place a clean nappy on the scales, zero the scales and remove the nappy, then weigh the infant while they are wearing a clean nappy of the same type that was used when the scales were zeroed.	Adults<br>1. Record the weight to the nearest 0.1 kg.<br>2. Repeat measures until two measurements are within 0.5 kg.<br>Infants<br>1. Record weight to two decimal places.
**Waist measures**	Equipment: Seca tape measure 201 1717009<br>1. It is preferable to measure next to the skin, however the measure can be taken over clothing if it is light and fitting.<br>2. Subject stands with feet close together, arms at the side and body weight evenly distributed.<br>3. Subject relaxed and the measurement is taken at the end of a normal expiration.<br>4. Measure the waist at a horizontal level 2.5 cm above the navel.		1. Record the measurement to the nearest 0.1 cm.<br>2. Repeat measures until two measurements are within 1.0 cm.
