# Supplementary material for: The Probiotics in Pregnancy Study (PiP Study): rationale and design of a double-blind randomised controlled trial to improve maternal health during pregnancy and prevent infant eczema and allergy
Source: BMC Pregnancy Childbirth. 2016 Jun 3;16:133. doi: 10.1186/s12884-016-0923-y (PMC4891898; doi:10.1186/s12884-016-0923-y)
Supplement: Additional file 3: Table S4. — Quality data for faecal samples. Description: list of quality control data recorded for infant and maternal faecal samples. (PDF 30 kb) [file 12884_2016_923_MOESM3_ESM.pdf]

Table 4: Quality data for faecal samples

Maternal Faecal Samples	Infant Faecal Samples
1. Date sample faecal sample collected.<br>2. Duration (in minutes) sample was kept at room temperature before being placed in home freezer: A: 0 - <5mins, B: 5 - <10mins, C: 10 - <15mins, D: ≥ 15 mins (specify).<br>3. Date sample placed in -80°C freezer.	1. Date and time faecal sample collected.<br>2. Whether sample was collected more than 10 mins after child defecated.<br>3. Whether the nappy was wet with urine at time of collection.<br>4. Duration (in minutes) sample was kept at room temperature before being placed in home freezer.<br>5. Date and time faecal sample was placed in -80°C freezer.
