# Supplementary material for: The Probiotics in Pregnancy Study (PiP Study): rationale and design of a double-blind randomised controlled trial to improve maternal health during pregnancy and prevent infant eczema and allergy
Source: BMC Pregnancy Childbirth. 2016 Jun 3;16:133. doi: 10.1186/s12884-016-0923-y (PMC4891898; doi:10.1186/s12884-016-0923-y)
Supplement: Additional file 4: Table S5. — Cord blood and tissue collection, processing and quality data. Description: Description of collection equipment, laboratory processing and quality control data for cord blood and tissue samples. (PDF 36 kb) [file 12884_2016_923_MOESM4_ESM.pdf]

Table 5: Cord blood and tissue collection, processing and quality data

	Cord Blood		Cord Tissue
**Sample**	6 ml cord blood	10-35 ml cord blood	2 x ½-1 cm sections cord tissue
**Sample collected into**	1 x 6 ml K2EDTA 10.8 mg tube (BD Diagnostics catalogue number 367873)	1 x 50 ml sterile light protected tube containing 20mls RPMI 1640 Medium (Gibco, 11875101 Life Technologies) and 750 units sodium heparin in saline with pH adjusted to 7.23 - 7.60 using sterile hydrochloric acid or sodium hydroxide if necessary	1 x 70 ml specimen jar (non-sterile) containing 20-30 ml Dulbecco's phosphate-buffered saline (DPBS Invitrogen Catalogue 14190-250)
**Transport**	On ice	Ambient	On ice or ambient
**Laboratory processing**	Centrifuge at 2200g for 10 min at 4°C<br>Aliquot plasma into 3 cryovials,<br>Mix remaining cell pallet and aliquot into 3 further cryovials<br>Freeze all at -80°C	Cord blood is processed using a protocol adapted from the 'BIO Cord Blood Processing – Double Layering' protocol from Childhood Allergy and Immunology Research (C.A.I.R.), School of Pediatrics and Child Health at the University of Western Australia<br>Plasma in RPMI is separated from the whole blood and frozen at -80°C<br>Red blood cells and granulocytes are frozen at -80°C<br>Mononuclear cells (monocytes and lymphocytes) are frozen at a concentration of 20 million per ml in liquid nitrogen	Drain off DPBS solution. Pour in more solution then drain off again<br>1 piece cord tissue in empty cryovial<br>2nd piece of cord tissue placed into another cryovial and sample covered with RNAlater® Stabilization Solution (Ambion, AM7020 Life Technologies)<br>Both samples stored at -80°C
**Quality data**	Date and time of sample collection		
	Time sample placed on ice		
	Date and time of arrival at laboratory		
	Confirmation sample arrives at laboratory on ice	Date and time processing began.<br>(Note samples more than 12 hours old or less than 10 ml of cord blood are discarded)	
	Date and time processed samples frozen		Date and time processed samples frozen
