# Supplementary material for: The Probiotics in Pregnancy Study (PiP Study): rationale and design of a double-blind randomised controlled trial to improve maternal health during pregnancy and prevent infant eczema and allergy
Source: BMC Pregnancy Childbirth. 2016 Jun 3;16:133. doi: 10.1186/s12884-016-0923-y (PMC4891898; doi:10.1186/s12884-016-0923-y)
Supplement: Additional file 5: Table S6. — Quality data for breast milk samples. Description: Details of data recorded for quality control of breast milk samples. (PDF 30 kb) [file 12884_2016_923_MOESM5_ESM.pdf]

Table 6: Quality data for breast milk samples

**Data completed by women**	1. Date and time sample collected<br>2. Sample collected from Right breast / Left Breast / Both Breasts (circle)<br>3. Time last fed from Left breast___:___ am/pm Right Breast___:___ am/pm
**Data collected by research staff**	1. At time of sample pickup, the sample was: frozen/chilled/fresh<br>2. Duration sample was at room temperature after the woman completed collecting the sample before it was chilled or frozen:<br>A: 0 - <5mins, B: 5 - <10mins, C: 10 - <15mins, D: ≥ 15 mins (specify)<br>3. Date and time sample stored at -80°C
